# Mammoth ivory was the most suitable osseous raw material for the production of Late Pleistocene big game projectile points

**DOI:** 10.1038/s41598-019-38779-1

**Published:** 2019-02-19

**Authors:** Sebastian J. Pfeifer, Wolfram L. Hartramph, Ralf-Dietrich Kahlke, Frank A. Müller

**Affiliations:** 1 Friedrich Schiller University Jena, Seminar Prehistoric Archaeology, Löbdergraben 24a, D-07743 Jena, Germany; 2 Friedrich Schiller University Jena, Otto Schott Institute of Materials Research, Löbdergraben 32, D-07743 Jena, Germany; 3 Senckenberg Research Institute, Research Station of Quaternary Palaeontology, Am Jakobskirchhof 4, D-99423 Weimar, Germany

## Abstract

Late Pleistocene societies throughout the northern hemisphere used mammoth and mastodon ivory not only for art and adornment, but also for tools, in particular projectile points. A comparative analysis of the mechanical properties of tusk dentine from woolly mammoth (*Mammuthus primigenius*) and African elephant (*Loxodonta africana*) reveals similar longitudinal stiffness values that are comparable to those of cervid antler compacta. The longitudinal bending strength and work of fracture of proboscidean ivory are very high owing to its substantial collagen content and specific microstructure. In permafrost, these properties can be fully retained for thousands of years. Owing to the unique combination of stiffness, toughness and size, ivory was obviously the most suitable osseous raw material for massive projectile points used in big game hunting.

## Introduction

Organic projectile technology for big game hunting is considered a crucial prerequisite for the human colonization of the northern hemisphere during the Pleistocene^1–5^.

In connection with the appearance of anatomically modern humans, there is an emphasis on the use of hard osseous tissues like cervid antler, large mammal bone and proboscidean ivory for the production of spear and lance heads^6^. Antlers from reindeer (*Rangifer tarandus*) and red deer (*Cervus elaphus*), which were easily accessible, easy to work and very tough^7–9^, were clearly the dominant raw material, especially during the Late Upper Palaeolithic in Europe^8,10–15^. However, ivory from woolly mammoth (*Mammuthus primigenius*) and from North American mastodon (*Mammut americanum*) also played an important role in various Late Pleistocene archaeological traditions throughout Europe^10,16–18^, Siberia^19,20^ and North America^21,22^.

**Figure 1:**
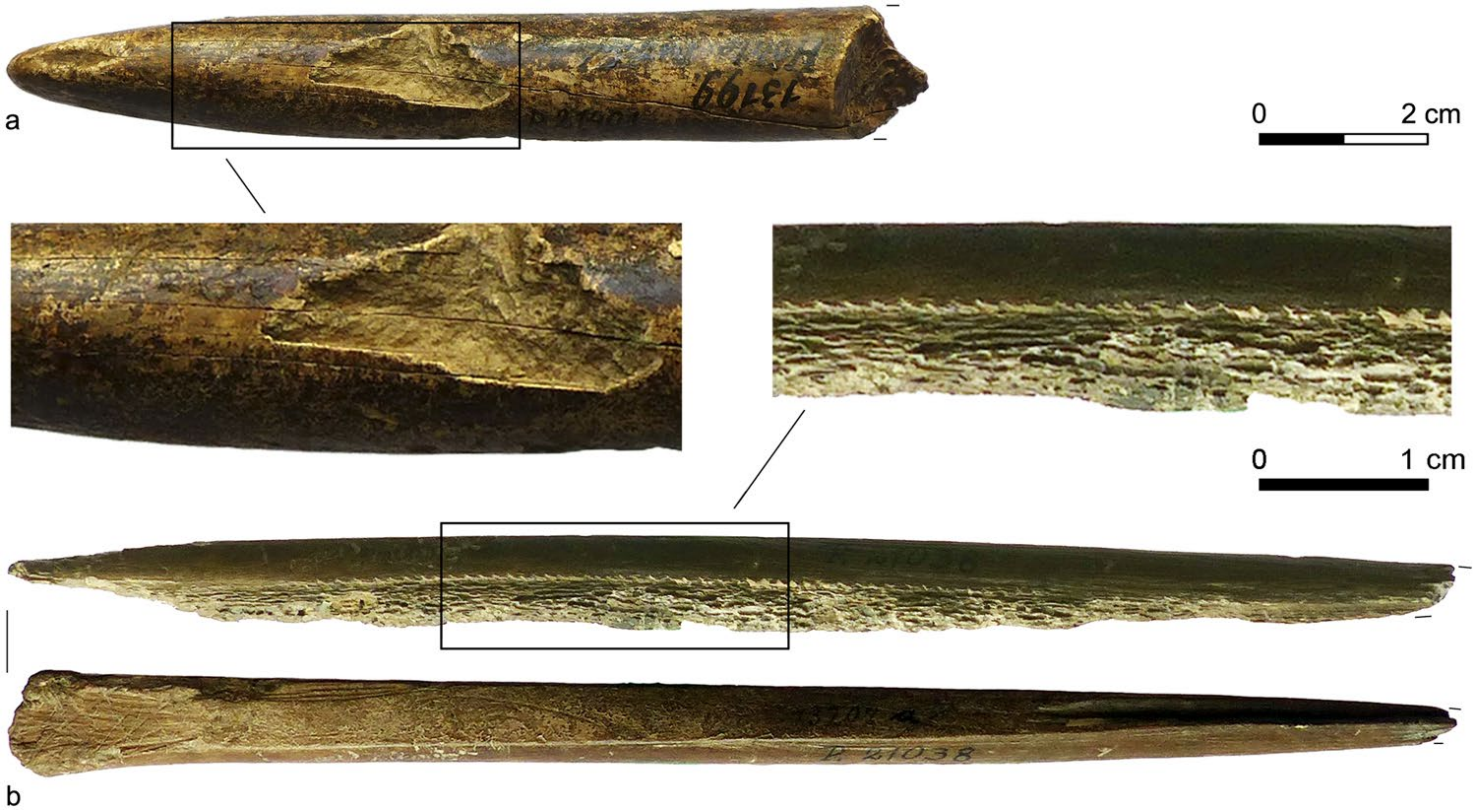
Late Upper Palaeolithic projectile points from the Pekárna cave site (Czech Republic). Collection Moravské Zemské Muzeum-Ústav Anthropos, Brno. (**a**) Medial-distal section of a mammoth ivory point (Inv.-No. P 21401). Note the lamellar structure due to the dentine cones. (**b**) Basal-distal section of an antler point (Inv.-No. P 21038). Note the compacta on the upper and the spongiosa on the lower side (Photographs S. J. Pfeifer).

But why was ivory used for projectiles? The procurement of mammoth tusks was associated with considerable effort or even risk^23–25^, the material is challenging to work with lithic tools^8,26,27^, and other suitable raw materials like antler and large mammal bone were always abundant. But even as woolly mammoths became increasingly rare and finally disappeared from Europe towards the end of the Late Glacial period^28,29^, their ivory which could be extracted from natural permafrost deposits, continued to be used by the societies of the Late Upper Palaeolithic for projectile points whenever it was available in sufficient size and quality (Fig. 1)^10,11,24,30^. Of course, the optical and haptic attractiveness of ivory is unsurpassed: No other biological raw material has such sublime colours and patterns and takes on such a smooth polish^7^. This is why mammoth ivory was preferred in humankind’s oldest portable artworks^23^. In a projectile point, however, favourable mechanical properties like hardness and stiffness for efficient energy transfer into the prey as well as toughness for fracture resistance are crucial^7,8,13,31^.

**Figure 2:**
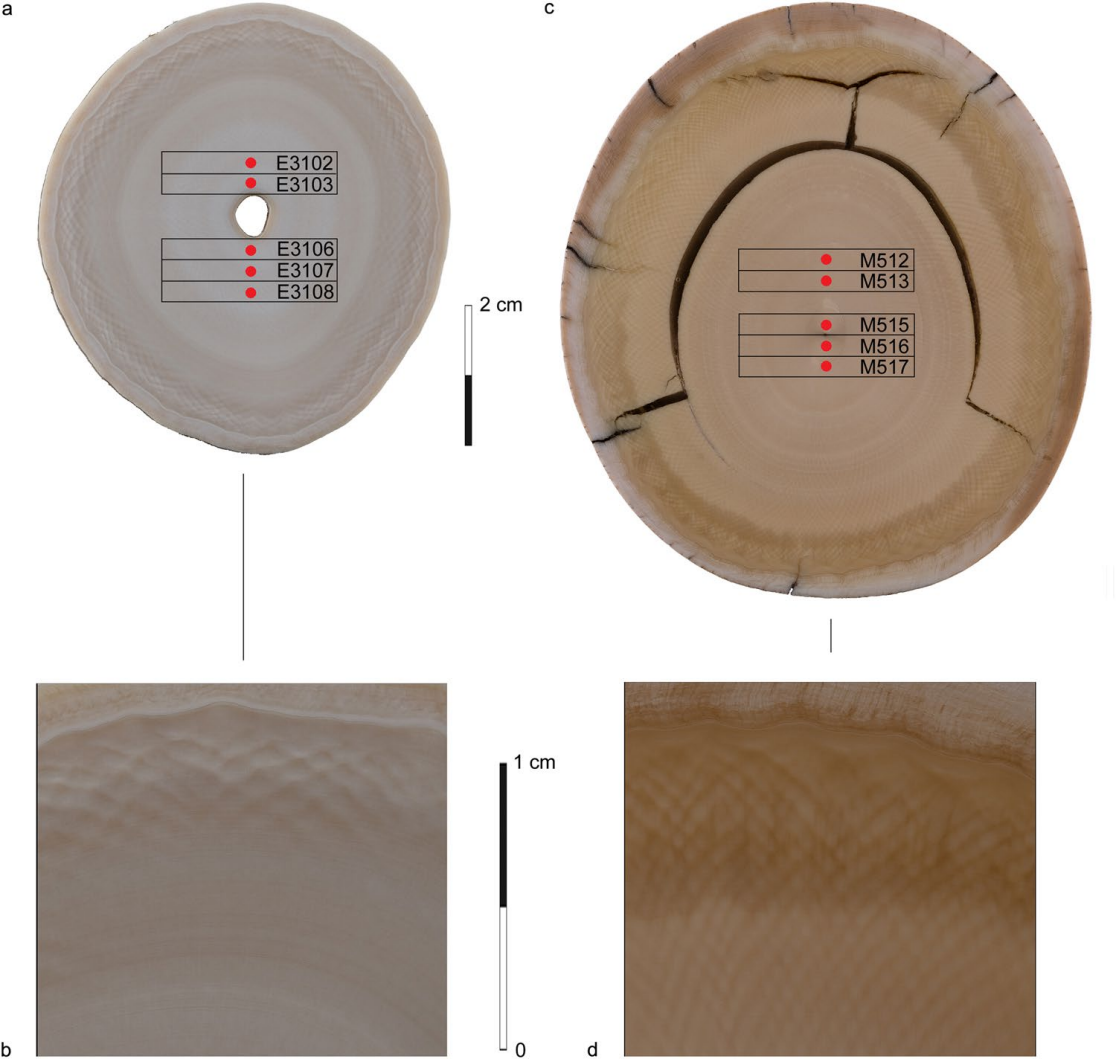
Cross sections of the tusk samples with positions of samples for XRD and positions of measurement (red dots) marked. (**a**,**b**) *Loxodonta africana*. Note the annual growth rings and the >90° angle of the Schreger pattern. (**c**,**d**) *Mammuthus primigenius*. Note the annual growth rings and the <90° angle of the Schreger pattern (Photographs S. Döring, Senckenberg Research Institute, Research Station of Quaternary Palaeontology Weimar).

The widespread use of proboscidean ivory in prehistoric tools indicates that it was a suitable raw material for mechanical applications^18,23^. But while there are numerous studies addressing the mechanical properties of antler and bone from different taxa (see Supplementary Table S2), corresponding information for ivory is very limited in the case of elephant and missing in the case of mammoth. In 1976^31^, the archaeologist Gerd Albrecht carried out a small series of mechanical tests on the material to investigate its usability for projectile points. He found that extant African elephant (*Loxodonta africana*) dentine, although very hard and stiff, was more fragile than antler and mammal long bone. These results, however, although widespread owing to a lack of recent studies^7,12,18^, are not in accordance with several ivory working experiments, which attest to both mammoth and elephant dentine having a considerable fracture toughness^23,32,33^. To counter this paradox and to explore how permafrost ivory compares to fresh material, new data on the chemical composition, microstructure and mechanical properties of tusk dentine from woolly mammoth (*M. primigenius*) and from African elephant (*L. africana*) (Fig. 2) were collected and subsequently compared with published information on antler and bone.

### Woolly mammoths and the biology of tusks

The genus *Mammuthus* Brookes, 1828 is assigned to the subfamily of modern elephants, Elephantinae Gray, 1821, along with the extant Asian and African elephants of the genera *Elephas* Linnaeus, 1758 and *Loxodonta* [Vigors], 1827 respectively. The woolly mammoth, *M. primigenius* Blumenbach, 1799, originated in NE Asia roughly 400 ka BP^34,35^. In Europe corresponding representatives of the mammoth evolutionary line first occurred much later, during the late Marine Isotope Stage (MIS) 7 or at the beginning of MIS 6, 200–160 ka BP^36,37^. Via Beringia *M. primigenius* reached northern North America during the Late Pleistocene^38^, where un-glaciated areas were occupied already during the Last Interglacial (MIS 5e, c. 123–110 ka)^39^. The Holarctic maximum distribution of fully developed woolly mammoths occurred during the last glacial period spanning MIS 5d-2, i.e., approximately the interval between 110 and 14 ka. The verifiable area of the Late Pleistocene range of *M. primigenius* at that time comprised some 33.301.000 square kilometers^37^.

Modern elephants have a pair of deciduous and permanent tusks which represent second upper incisors. In woolly mammoth, as in all members of the mammoth evolutionary line both sexes grow tusks. New-born calves have 4–7 cm long deciduous tusks, crowned with a thin layer of enamel. During the first year of the animals’ life, non-enameled tusks replace these milk incisors^40^. The rootless permanent mammoth tusks grow throughout life. They are strictly structured by a succession of dentine cones. Each of these several millimetres to some centimetres thick and up to 35 cm long cones represents the growth progress of one single year. The dentine layers are built up in the tusk pulp cavity, i.e., the most proximal dentine cone is the youngest one. The nutrient- and mineral-rich spring and especially summer months lead to a significant increase of dentine and thereby of the tusks’ length and proximal circumference. During the fall, the growth rate is reduced to come to an end towards the end of the winter. A new growth period begins next spring. The resulting winter / spring discontinuity forms a sharp interface between the dentin cones. Oxygen isotope profiles confirm the annual nature of these cycles^41^. The growth history of tusks provides important information about the individual life history of mammoths^42^.

Tusks of *M. primigenius*, especially those of the relatively small Late Pleistocene forms, are mostly larger in relation to the animals’ body size compared to that of extant Asian and African elephants. Corresponding mechanical features of the ivory (see below) enable such an impressive growth. A stable fixation of the heavy mammoth tusks in the skull is achieved by a strong torsion of the former. The appearance of the teeth is determined by size and shape of the alveolar cavities which contain about one third of the tusks. Changes in alveolar morphology during individual growth of the skull are reflected in the resulting torsion patterns of the tusk.

Male tusks are mostly larger than those of same-age females. The growth pattern of the thinner and less curved female teeth slowed down from their first maternity onwards^40^. As male mammoths, similarly to male extant Asian and African elephants, probably segregated themselves from females over long periods of the year, there was little selective pressure for the latter to increase the size of their tusks^43^. The tusks of old mammoth bulls can reach lengths of up to 3.5 m and more (measured along the largest radius of the curvature), and weights up to 90–100 kg^40,43^. Garutt^44^ refers one isolated case of a couple of fossil tusks from the Indigirka River (Yakutia), each weighing some 150 kg. Observations on African elephant populations show that there is no linear correlation between tusk size and individual age; animals of the same age and sex can develop very different tusk dimensions^45,46^.

The original purpose for the evolution of elephantine tusks was presumably intra-specific contest. The size of the teeth signalises the individual status within a group^47^. Well-developed tusks achieve dominance during feeding and drinking competition^43^ and also act as a threat and weapon in inter-specific conflicts. Observations on extant Asian and African elephants prove, that these animals also use their incisors to gather food by pushing trees down, lifting up rootage or by stripping bark^43^. The leverage put into action on such occasions can lead to breakage or even to complete loss of tusks, as the existence of recent and fossil “Ganesha” elephants, especially in species with less twisted tusks, testifies^48^. Splintering of the tusk tips occurs also when the animals dig to loosen mineral-rich subsoil^43^. Elephants have a high individual need especially for calcium and sodium. Rough estimations for requirements of Asian elephants assume up to 60 g Ca and up to 100 g Na per day^49^. Such quantities might have been exceeded by *M. primigenius* owing to the growth of their larger sized tusks.

**Figure 3:**
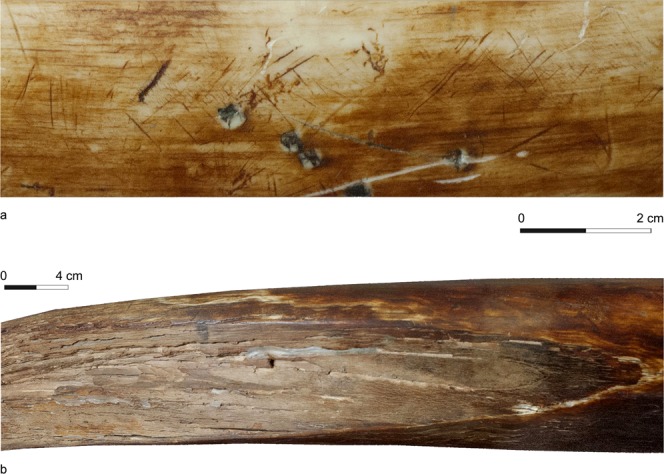
Left male tusk of Late Pleistocene *Mammuthus primigenius* from the Sundrun river valley (west of the Alazeja river), 5 km upstream of its mouth to the East Siberian Sea, Kolyma lowlands, NE Republic of Sakha (Yakutia), Russia; collection National Alliance of Shidlovskiy “Ice Age” Moscow, without inventory number. Indications of repeated similar use of the tooth during the lifetime of the individual: (**a**) Outer curvature in the middle section of the tusk with sharp, partially crossed scratches (dark) of c. 1–2 cm length besides recent damage in the form of elongated scratches (light) and traces of pickaxe (quadrangular). (**b**) Extended, flat wear facet at the outer curvature of the distal section of the tusk (Photographs I. Kirillova).

The curved incisors of mammoths were less suitable as levers than the tusks of forest or savannah elephants. However, not uncommon sharply deepened scratches and/or extended flat wear facets especially at the outer curvature of the tusks (Fig. 3)^45,50^ indicate regular and vigorous ground or ice contact of many mammoth tusks. Probably the animals occasionally moved frozen sediment and wind-hardened or refrozen snow, to prepare and facilitate the taking in of available vegetation of herbs and bushes. The heavy teeth were certainly also used to smash up the ice of frozen-over watering places^51^. It seems likely that during all these actions the strongest load was applied to the curved under (respectively outer) side of the teeth.

## Results

### Chemical composition and structure

Demineralization and deproteinization of tusk samples reveal that both woolly mammoth and African elephant dentine contain mineral and organic phases in similar proportions. According to our experiments the mineral phase is 59.3 ± 0.4 mass-% for mammoth and 59.4 ± 0.8 mass-% for elephant. The protein content reaches 33.6 ± 0.7 mass-% and 33.4 ± 0.8 mass-%, respectively. The missing proportion of approx. 7 mass-% can be assigned to the water that evaporated during the drying process. XRD results show that the main crystalline component of proboscidean dentine is hydroxyapatite (see Supplementary Figs S4 and S5). Crystalline domain sizes in the c-direction of apatite were calculated from the (002) peak broadening at 26° using equation (1), and are 21.4 ± 0.4 nm for *M. primigenius* and 23.0 ± 1.4 nm for *L. africana*. The intensity ratio I(002)/I(211) of 0.8 for transversal sections of both mammoth and elephant specimens indicates a slight c-axis orientation of the apatite crystals along the tusk axis. The ICP-OES results show that the apatite crystals in both kind of tusks are calcium deficient when compared to stoichiometric apatite (Ca_10_(PO_4_)_6_(OH)_2_: Ca/P = 1.67) and are substituted by magnesium and sodium ions in a considerable amount (Table 1).

**Table 1:** ICP-OES results.

	Mammuthus primigenius	Loxodonta africana
Ca/P	1.35 ± 0.004	1.27 ± 0.004
(Ca + Mg)/P	1.59 ± 0.004	1.51 ± 0.003
(Ca + Na)/P	1.41 ± 0.004	1.34 ± 0.004
(Ca + Mg + Na)/P	1.64 ± 0.003	1.58 ± 0.003

### Mechanical properties

**Figure 4:**
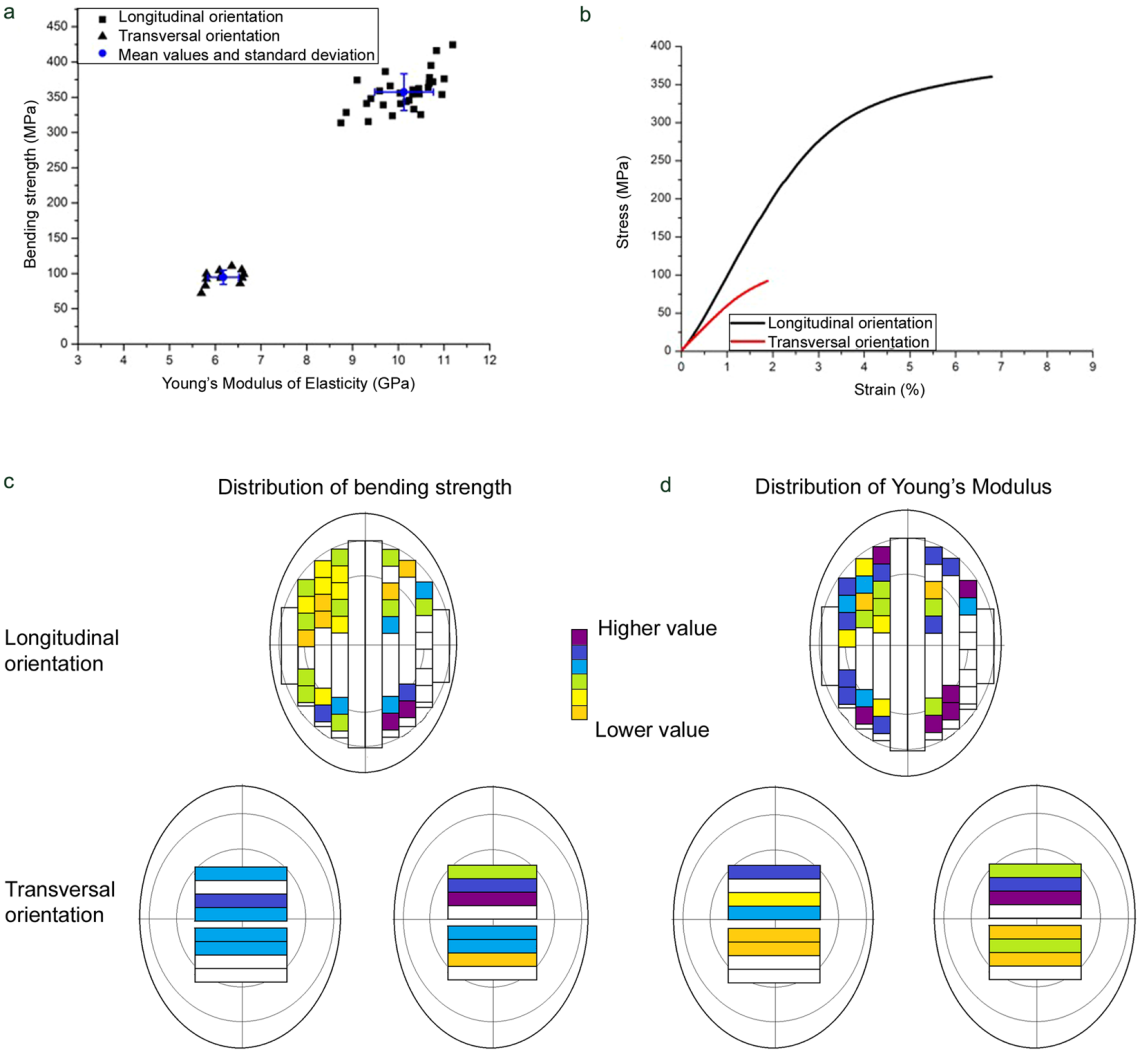
Mechanical properties of *Mammuthus primigenius* tusk dentine. (**a**) Bending strength and Young’s modulus for longitudinal and transversal samples. (**b**) Stress-strain diagram for longitudinal and transversal samples. (**c**) Gradients of the bending strength for longitudinal and transversal samples as a function of their position within the tusk. No scale. (**d**) Gradients of Young’s modulus for longitudinal and transversal samples as a function of their position within the tusk. No scale.

**Figure 5:**
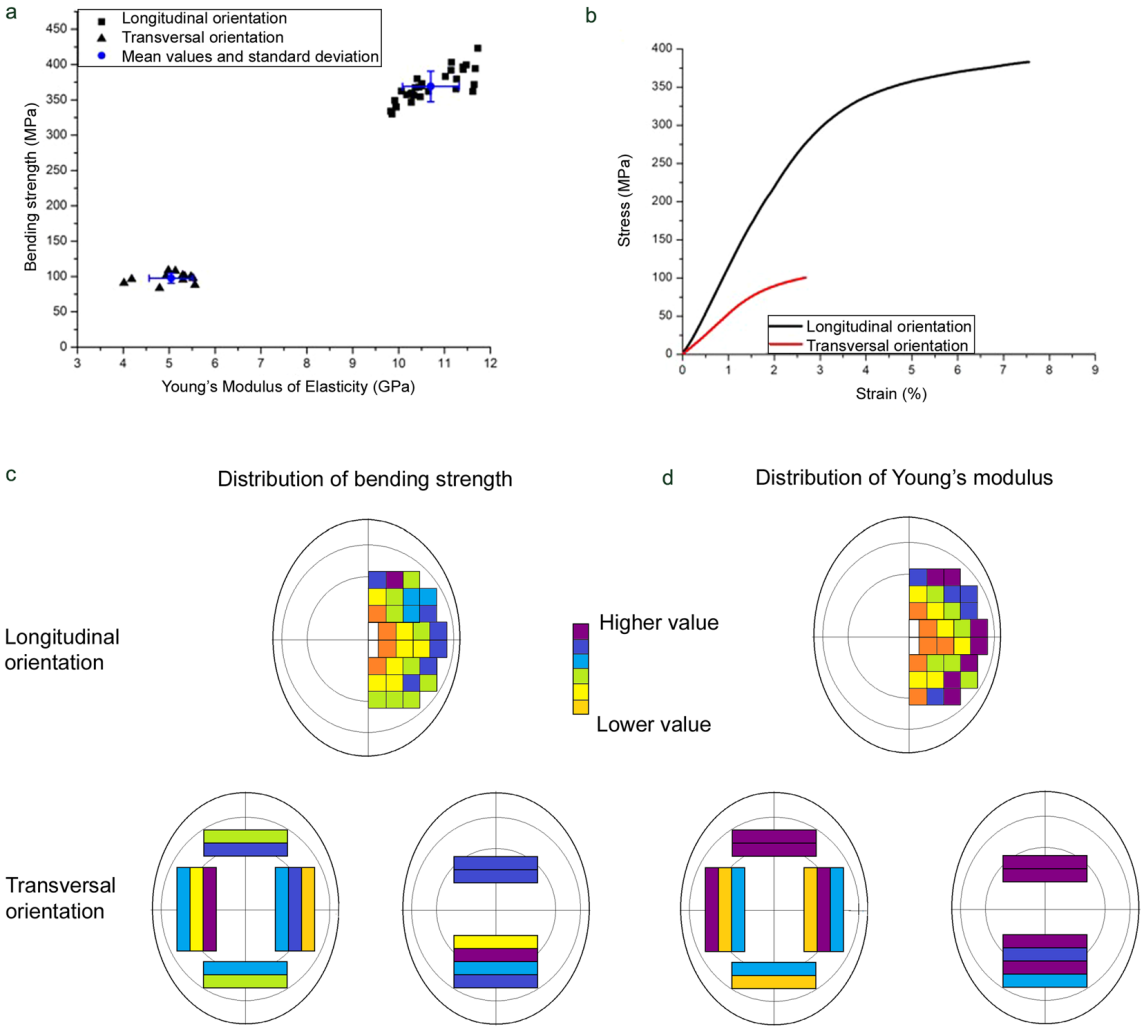
Mechanical properties of *Loxodonta africana* tusk dentine. (**a**) Bending strength and Young’s modulus for longitudinal and transversal samples. (**b**) Stress-strain diagram for longitudinal and transversal samples. (**c**) Gradients of the bending strength for longitudinal and transversal samples as a function of their position within the tusk. No scale. (**d**) Gradients of Young’s modulus for longitudinal and transversal samples as a function of their position within the tusk. No scale.

Figures 4 and 5 and Table 2 show the results of the 3-point bending tests. Again, woolly mammoth and African elephant exhibit very similar properties. In the longitudinal direction Young’s Modulus of Elasticity, a measure for the stiffness of a material, is 10.1 ± 0.6 GPa for mammoth and 10.7 ± 0.6 GPa for elephant dentine. The bending strengths are 357.3 ± 26.1 MPa and 369.0 ± 21.8 MPa, respectively. The work of fracture, a measure of the damage tolerance and toughness of a material, is 22.3 ± 10.0 kJ/m² for mammoth and 23.8 ± 6.9 kJ/m² for elephant. In the transversal direction, the mechanical properties are significantly reduced and the Young’s modulus and work of fracture are different for mammoth and elephant. The Young’s modulus is 6.2 ± 0.3 GPa and 5.0 ± 0.5 GPa, and the work of fracture is 0.4 ± 0.2 kJ/m² and 1.1 ± 0.5 kJ/m², respectively. The bending strengths are similar and amount to 94.9 ± 10.7 MPa for mammoth and 97.0 ± 6.4 MPa for elephant (Figs 4a,b and 5a,b).

**Table 2:** Mechanical properties of *Mammuthus primigenius* and *Loxodonta africana* tusk dentine.

		Mammuthus primigenius	Loxodonta africana
3-point bending test, longitudinal orientation	Young’s modulus (GPa) Bending strength (MPa) Work of fracture (kJ/m²)	10.1 ± 0.6 357.3 ± 26.1 22.3 ± 10.0	10.7 ± 0.6 369.0 ± 21.8 23.8 ± 6.9
3-point bending test, transversal orientation	Young’s modulus (GPa) Bending strength (MPa) Work of fracture (kJ/m²)	6.2 ± 0.3 94.9 ± 10.7 0.4 ± 0.2	5.0 ± 0.5 97.0 ± 6.4 1.1 ± 0.5
Compression test, longitudinal orientation	Compression modulus (GPa)	4.7 ± 0.3	4.4 ± 0.5
Indentation test	Vickers hardness H_V_0.1 (GPa)	35.2 ± 1.2	41.2 ± 1.0

Additionally, the spatial distribution of both bending strength and Young’s modulus was determined for both orientations as shown in Figs 4c,d and 5c,d. For the longitudinal samples both properties appear to form a gradient with a slight decrease towards the central pulp cavity. Because of the smaller sample size, it is more difficult to see a trend in the distribution for the transversal samples, but there also appears to be a gradient, this time with decreasing values from the inside of the tusk to the outside.

Compression tests of longitudinal sections show that the compression moduli of mammoth and elephant are very similar at 4.7 ± 0.3 GPa and 4.4 ± 0.5 GPa, respectively. The results of the Vickers hardness tests indicate a significant difference in the hardness of the two types of tusks. The mean Vickers hardness (H_V_0.1) for mammoth dentine is 35.2 ± 1.2 MPa while elephant dentine has a hardness of 41.2 ± 1.0 MPa.

## Discussion

**Figure 6:**
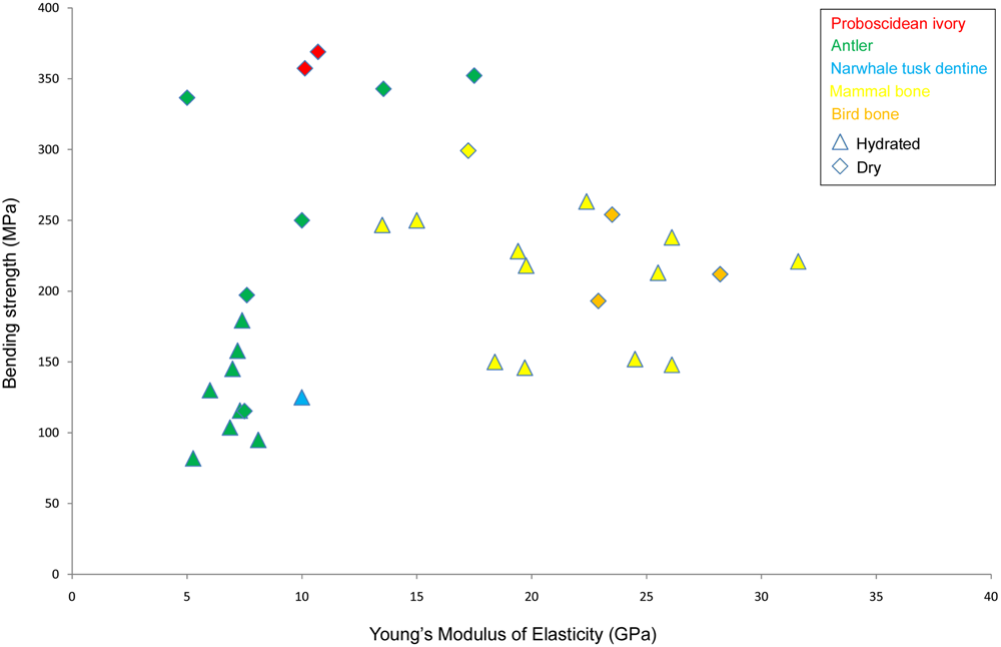
Longitudinal bending strength and Young’s modulus of selected osseous tissues. References can be found in the Supplementary Table S2.

Figure 6 and online Supplementary Table S2 show the mechanical properties of selected vertebrate skeletal and dental elements known from the literature. It is striking that the values themselves are very heterogeneous, even within the same species. In the case of reindeer antler compacta with a relatively coherent modulus of elasticity between 5 and 8 GPa, for example, the bending strength varies by over 300%. Reasons for this can be the moisture content of the tested samples (fresh, soaked or dry), differing experimental set-ups and inherent variations in a biological material. In any case, there is a clear trend: antler is not as stiff as terrestrial mammal and bird bone, but it is harder to break. This is owing to antler’s lower mineralization^52^. The examined proboscidean dentine, both from permafrost and extant, displays longitudinal stiffness values which are quite high at 10 GPa considering its organic content of 34 mass-%. For example, the compacta of undomesticated barren-ground caribou (*R. tarandus groenlandicus*) was previously tested to 5 GPa applying exactly the same test protocol (see Supplementary References S3). At the same time, ivory corresponds to the highest values for bending strength and work of fracture of reindeer and red deer (*C. elaphus*) antler which is known for its great toughness. Soaked dentine from narwhale (*Monodon monoceros*) tusk has the same stiffness as dry proboscidean dentine, but is only about 1/3 as strong.

The paradoxical combination of high stiffness, strength and toughness in mammoth and elephant dentine can be explained in particular by its hierarchically oriented microstructure in combination with its high organic content, which essentially consists of the protein collagen^53^. It is well known that biominerals with a hierarchical organization of their nanoscale microstructure have outstanding mechanical properties in terms of strength and toughness^54–56^. In the event of a damaging impact, a crack propagates at the interface between the inorganic and organic phase where the specific nano-architecture of the biomineral leads to a significant reduction of crack tip energy owing to energy-dissipating processes such as crack deflection, tilting and twisting^57^. Prominent examples of such biominerals are nacre^58^, conch shell^59^, tooth of marine mollusks^60^, and dermal armour^61^.

As with other osseous tissues, proboscidean ivory shows a hierarchical organization on the nanoscale resulting from the specific alignment of apatite crystals along collagen fibrils^62^. Beyond that, the tusks display some unique micro-structural features such as concentric cones that occur during specific growth periods (see above) and a Schreger pattern^63^ formed by a network of microtubuli that perforate the ivory matrix. In contrast to the parallel alignment of the dentine tubuli in mammalian tooth, the microtubuli in proboscidean tusks are aligned sinusoidal^64^. They form a cross-hatched, helical network of intersecting Schreger lines that is visible to the naked eye. The resulting Schreger angles characterize different evolutionary lines of proboscideans, and can thus help to distinguish between recent and fossil ivory^65^: Schreger angles considerably above 90° are reported for African elephant while for woolly mammoth, they are slightly below 90° (Fig. 2b,d)^66^. This specific orientation also explains differences in the orientation of the apatite crystals in long mammalian bones compared to proboscidean tusk. While the I(002)/I(211) ratio of a hydroxyapatite powder sample is 0.5, it amounts up to 3.4 in long bone^67^. This high ratio indicates a preferred growth orientation of the apatite crystals, whose c-axis is aligned parallel to the collagen fibres in the longitudinal direction of the bone. On the other hand, the intensity ratio of proboscidean tusks is only 0.8, indicating that neither the collagen fibrils nor the apatite crystals are preferentially aligned in the tusk axis, but in a more complex manner following the sinusoidal orientation of the microtubuli. This specific orientation of the microtubuli in proboscidean ivory leads to the observed differences of the mechanical properties in longitudinal and transversal direction, since the majority of elongated helical pores are aligned in the radial direction^64^ and thus facilitate crack propagation. Whereas in longitudinal samples, no significant differences between woolly mammoth and African elephant can be found, in transversal samples, a significantly reduced stiffness but higher work of fracture and hardness for African elephant compared to woolly mammoth are revealed. This indicates that differences in the Schreger angles resulting from a different orientation of the microtubuli (Fig. 2) only have a negligible effect on the mechanical properties when force is applied perpendicular to growth direction but quite a significant one when it is applied parallel to it.

The slightly higher values for longitudinal bending strength and modulus of elasticity in the outer areas of the tusks from both mammoth and elephant might reflect the pattern of mechanical stress caused by their natural use during the animal’s life time (see above).

## Conclusion

Proboscidean ivory was an excellent raw material for Late Pleistocene osseous projectile points. Concerning bending strength and work of fracture, mammoth and elephant dentine are comparable to the tough compacta of cervid antler. Thus, corresponding ivory specimens shared their high resilience and durability. In terms of stiffness, proboscidean dentine is superior to reindeer antler, but in some cases can be surpassed by red deer antler. Thanks to its structure, however, it allows the production of completely compact workpieces (Fig. 1a), while antler projectiles of similar size must always consist of a substantial proportion of spongy tissue (Fig. 1b)^13^. Since strength and stiffness are functions of density, the solid ivory projectile is therefore more robust and stiffer than its partially porous antler counterpart. Moreover, with their large dimensions, proboscidean tusks are predestinated for the manufacture of very large projectiles, especially in conditions where suitable timber is scarce or absent^25,68^. These unique properties suggest that ivory was the best available osseous raw material for making massive spear and lance heads that could be used to hunt reindeer/caribou (*R. tarandus*), horse (*Equus*), bison (*Bison priscus*), brown and cave bear (*Ursus arctos*, *U. spelaeus*) and, of course, woolly mammoth^10,25,69–72^. After the regional disappearance of mammoths towards the end of the Pleistocene, their ivory could still be transported from other areas or collected from natural deposits and processed into projectile points of striking appearance and lethal efficiency. Permafrost ivory can not only have the same mechanical properties as fresh material, but was also worked more easily owing to its lower hardness, especially with the groove-and-splinter technique that is typical of Late Upper Palaeolithic projectile production^73^.

## Methods

The experiments used tusks from woolly mammoth from permafrost and extant African elephant (Fig. 2). Both were purchased from the professional ivory carver and authorized dealer Jürgen Schott (Erbach, Germany). The elephant samples come from the left tusk of *Loxodonta africana* (Cites certification number: DE-122/14 + 2,650 kg). The mammoth samples were taken from a well-preserved Siberian tusk of *M. primigenius*. Both tusk sections represent seven growth cycles.

To determine the differences in chemical composition and microstructure of mammoth and elephant ivory XRD, ICP-OES, SEM, deproteinization and experimental demineralisation were performed. For XRD, CuKα radiation (λ = 1.5406 Å) and measurement time of one hour per sample were used (2 Theta range 15–72°, step size 0,005°, scan rate c. 1°/min). For each kind of tusk, five samples taken from similar relative positions were measured in the tusk growth direction (Fig. 2a,c). The crystal domain size τ in direction of the crystallographic c-axis was calculated using the Scherrer equation (1)

1 $$\tau =\frac{K\,\lambda }{\beta \,cos\theta }$$

with the shape factor K which was assumed as 0.9, the wavelength λ, the FHWM and the Bragg angle θ of the (002) peak at 2θ = 26°. For each tusk, the mean value, standard deviation and variance were determined (Supplementary Table S6).

ICP-OES samples were prepared by dissolving 50 mg ivory powder in 2 ml of concentrated nitric acid (65 mass-%) for two hours. After dissolving, 8 ml of pure water were added.

For demineralization, samples with a volume of approx. 300 mm³ were stored in 0.6 M hydrochloric acid. The acid solution was replaced every 24 hours. After seven days, the samples were washed with de-ionized water and freeze-dried for another 24 hours. For deproteinization, samples with a volume of approx. 300 mm³ were kept in a 2.17 wt% sodium hypochlorite solution for 14 days, which was replaced every 24 hours. The samples were then washed with de-ionized water and dried for 24 hours.

Specimens for 3-point bending tests and compression tests had dimensions of 3 mm*4 mm*25 mm and 5 mm*5 mm*10 mm, respectively. The moisture content was 7 mass-%. Samples were taken in two different orientations, longitudinal (in growth direction of the tusk) and transversal (perpendicular to the growth direction). To ensure exact orientations, the samples were extracted from 30 mm thick slices cut perpendicular to the pulp cavity (Figs 2, 4c,d and 5c,d).

For 3-point bending tests, 30 longitudinal and 12 transversal samples were used. The samples were cut with a carbide saw blade from the dentine part of the tusks. The samples were then polished before the mechanical test using 1200 grit coated abrasives. Both sawing and polishing were carried out at low speed to avoid dehydration and collagen degradation. The tests were conducted at room temperature on a Zwick/Roell Z020 machine. The distance between the sample carriers was 16 mm. The pre-load was 0.5 N and loading speed 2 mm/min. The breaking point was determined by the total failure of the samples defined by a 20% decrease in the test load.

To determine the hardness of the ivory, Vickers hardness tests were conducted on polished samples parallel to growth direction with a Vickers pyramid and a Shimadzu HMV-2000 machine. The load was 100 g and load duration was 10 seconds. Twenty measurements were carried out in each case.

## Supplementary information

Supplementary Information - Mammoth ivory was the most suitable osseous raw material for the production of Late Pleistocene big game projectile points

## Data Availability

All data generated or analysed during this study are included in this published article (and its online Supplementary Information files). The samples on which the 3-point bending tests and compression tests were carried out are archived at the Senckenberg Research Station of Quaternary Palaeontology in Weimar, Germany. *Mammuthus primigenius*: IQW 2018/45 415 (Sibirien 50 724), IQW 2018/45 416 (Sibirien 50 725), IQW 2018/45 417 (Sibirien 50 726). *Loxodonta africana*: IQW 2018/45 418 (Afrika 50 727), IQW 2018/45 419 (Afrika 50 728), IQW 2018/45 420 (Afrika 50 729).
