# Supplementary material for: Mammoth ivory was the most suitable osseous raw material for the production of Late Pleistocene big game projectile points
Source: Sci Rep. 2019 Feb 19;9:2303. doi: 10.1038/s41598-019-38779-1 (PMC6381109; doi:10.1038/s41598-019-38779-1)
Supplement: Supplementary file 1 — Supplementary Information - Mammoth ivory was the most suitable osseous raw material for the production of Late Pleistocene big game projectile points [file 41598_2019_38779_MOESM1_ESM.pdf]

Supplementary Information

Mammoth ivory was the most suitable osseous raw material for the production of Late Pleistocene big game projectile points

Sebastian J. Pfeifer*
Friedrich Schiller University Jena
Seminar Prehistoric Archaeology
Löbdergraben 24a, DE-07743 Jena
sebastian.pfeifer@uni-jena.de

Wolfram L. Hartramph
Friedrich Schiller University Jena
Otto Schott Institute of Materials Research
Löbdergraben 32, DE-07743 Jena

Ralf-Dietrich Kahlke
Senckenberg Research Station of Quaternary Palaeontology
Am Jakobskirchhof 4, DE-99423 Weimar

Frank A. Müller
Friedrich Schiller University Jena
Otto Schott Institute of Materials Research
Löbdergraben 32, DE-07743 Jena

Supplementary Table S1: Measured values of Young's Modulus of Elasticity and longitudinal bending strength for mammoth (*M. primigenius*) and African elephant (*L. africana*) dentine.

$E_{mammoth}$ longitudinal (GPa)	$\sigma_{mammoth}$ longitudinal (MPa)	$E_{mammoth}$ transversal (GPa)	$\sigma_{mammoth}$ transversal (MPa)	$E_{elephant}$ longitudinal (GPa)	$\sigma_{elephant}$ longitudinal (MPa)	$E_{elephant}$ transversal (GPa)	$\sigma_{elephant}$ transversal (MPa)
11.0	353.7	5.8	92.3	11.1	391.9	5.5	100.1
10.3	333.1	6.1	93.7	11.7	423.0	5.3	100.5
9.7	339.1	5.8	99.7	11.6	361.9	5.6	88.1
9.6	358.9	6.1	96.3	10.3	356.3	5.1	108.0
9.3	341.0	6.5	86.0	10.5	369.8	5.3	95.5
9.1	374.4	6.6	93.9	11.3	379.6	4.9	100.5
10.5	354.6	6.6	99.1	11.0	383.2	5.5	97.7
9.4	348.2	6.6	105.5	9.9	339.7	4.0	90.9
10.2	345.5	6.4	110.6	10.4	367.4	5.0	108.7
8.7	313.6	6.1	104.5	10.4	379.7	4.2	96.2
9.9	323.7	5.7	72.1	11.2	403.0	5.3	101.8
10.1	340.8	5.8	82.7	9.9	340.2	4.8	83.6
10.8	371.9			10.2	357.0		
10.3	354.3			10.5	373.0		
10.2	343.6			11.4	392.9		
10.5	362.3			9.8	333.7		
9.3	315.4			9.9	349.0		
10.7	363.9			10.3	359.0		
10.7	369.6			11.5	399.5		
10.3	360.4			9.9	329.8		
8.9	328.4			10.5	354.2		
9.8	366.0			10.4	368.0		
10.7	377.8			11.4	396.7		
9.7	386.4			10.3	359.5		
10.8	416.0			10.3	346.5		
10.5	325.2			11.7	394.2		
10.7	395.1			10.7	361.9		
11.2	424.3			10.1	362.2		
11.0	376.1			11.3	365.6		
10.0	355.9			11.6	371.8		

Supplementary Table S2: Published experimental data for Young's Modulus of Elasticity, longitudinal bending strength and work of fracture of selected osseous tissues.

**Material tested**	**Sample size**	**Young's Modulus of elasticity [GPa]**	**Bending strength [MPa]**	**Work of fracture**	**Reference**
Mammuthus primigenius - dry dentine	30	10.1 ± 0.6	357.3 ± 26.1	22.3 ± 10.0 kJ/m^2	This work
Loxodonta africana - dry dentine	30	10.7 ± 0.6	369.0 ± 21.8	23.8 ± 6.9 kJ/m^2	This work
Rangifer tarandus groenlandicus - dry antler	?	5.0 ± 1.0	336.6 ± 43	-	S3: I
Monodon monoceros - soaked dentine	56	10.0	125	-	S3: II
Axis axis - dry femur	?	31.6	221	-	S3: III
Rangifer tarandus - soaked antler	6	8.1	95	32 MJ m^{-3}	S3: IV
Rangifer tarandus - soaked antler	26	5.8 ± 0.4	-	-	S3: V
Rangifer tarandus - soaked antler	4	6.4 ± 1.7	-	-	S3: VI
Rangifer tarandus - soaked antler	5	6.0 ± 1.0	130 ± 20	18 ± 4 kJ/m^2	S3: VII
Alces alces - soaked antler	36	11.6 ± 45	-	-	S3: VIII
Cervus canadensis - dry antler	12	7.6 ± 0.25	197.3 ± 24	-	S3: IX
Cervus canadensis - soaked antler	12	6.98 ± 0.26	145.1 ± 9	-	S3: IX
Cervus elaphus - soaked antler	?	5.27 ± 0.33	81.9 ± 4.7	18.22 ± 0.91 kJ/m^2	S3: X
Cervus elaphus - soaked antler	?	6.87 ± 0.28	103.7 ± 3.9	22.24 ± 0.84 kJ/m^2	S3: X
Cervus elaphus - soaked antler	?	7.4 ± 0.7	179.4	6.186 kJ/m^2	S3: XI
Cervus elaphus - soaked antler	8	6.71 ± 5	-	-	S3: VI
Bos taurus - dry tibia	5	17.24	299.2	6.468 kJ/m^2	S3: XII
Cervus elaphus - dry antler	5	13.55	342.8	17.54 kJ/m^2	S3: XII
Cervus elaphus - soaked antler	13	7.2	158	93 MJ m^{-3}	S3: IV
Cervus elaphus - dry antler	?	10	250	-	S3: III
Cervus canadensis - dry antler	7	7.5	115.4 ± 16.6	-	S3: XIII
Cervus elaphus - dry antler	25	17.5 ± 0.5	352.2 ± 8.8	23.4 ± 1 kJ/m^2	S3: XIV

Cervus elaphus<br>- soaked antler	25	7.30 ± 0.3	115.7 ± 3.7	31.0 ± 1.3<br>kJ/m^2	S3: XIV
Cervus elaphus<br>- soaked femur	24	22.39 ± 0.33	263.3 ± 5.5	9.6 ± 0.3<br>kJ/m^2	S3: XIV
Cervus elaphus<br>- fresh femur	2	15	250	11<br>kJ/m^2	S3: VII
Dama dama<br>- soaked radius	4	25.5	213	21<br>MJ m^{-3}	S3: IV
Capreolus capreolus<br>- soaked femur	3	18.4	150	3<br>MJ m^{-3}	S3: IV
Odocoileus virginianus<br>*- fresh radius*	6	10 ± 2	170 ± 30	17<br>kJ/m^2	S3: VII
Bos taurus<br>- soaked femur	4	26.1	148	4<br>MJ m^{-3}	S3: IV
Bos taurus<br>- soaked tibia	4	19.7	146	4<br>MJ m^{-3}	S3: IV
Bos taurus<br>- soaked femur	?	13.5	246.7	1.71<br>kJ/m^2	S3: XI
Bos taurus<br>- fresh femur	10	19.41	228.2	25.1<br>kJ/m^2	S3: XV
Bos taurus<br>- fresh femur	?	26.1	238	-	S3: XVI
Bos taurus<br>- soaked femur	10	19.76	218	30.3<br>kJ/m^2	S3: XV
Equus caballus<br>- soaked femur	4	21.22 ± 1.9	-	-	S3: VI
Equus caballus<br>- soaked femur	2	24.5	152	5<br>MJ m^{-3}	S3: IV
Phoenicopterus sp.<br>- dry tibiotarsus	?	28.2	212	-	S3: III
Grus antigone<br>- dry tibiotarsus	?	23.5	254	-	S3: III
Aptenodytes patagonicus<br>- dry ulna	?	22.9	193	-	S3: III

Supplementary References S3

I. Krey, J., Charakterisierung der Materialeigenschaften des traditionellen Werkstoffs Rengeweih in Hinblick auf prähistorische Artefakte. Bachelor thesis, Friedrich Schiller University Jena (2013).

II. Currey, J. D., Brear, K., Zioupos, P. Dependence of mechanical properties on fibre angle in narwhale tusk, a highly oriented biological composite. *J. Biomech.* **27,** 885–897 (1994).

III. Currey, J. D., The design of mineralised hard tissues for their mechanical functions, *J. Exp. Biology* **202,** 3285–3294 (1999).

IV. Currey, J. D., Physical characteristics affecting the tensile failure properties of compact bone, *J. Biomech.* **23,** 837–844 (1990).

V. Shah, S. R., Desjardins, J. D., Blob, R. W. Antler stiffness in caribou (*Rangifer tarandus*): testing variation in bone material properties between males and females. *Zoology* **111,** 476–482, doi: 10.1016/j.zool.2007.12.001 (2008).

VI. Currey, J. D., The effect of porosity and mineral content on the Young's Modulus of Elasticity of compact bone. *J. Biomech.* **21,** 131–139 (1988).

VII. Margaris, A. V. The mechanical properties of marine and terrestrial skeletal materials. *Ethnoarchaeology* **1,** 163–184, doi: 10.1179/eth.2009.1.2.163 (2009).

VIII. Blob, R. W. & Snelgrove, J. M. Antler stiffness in moose (*Alces alces*): correlated evolution of bone function and material properties? *J. Morphol.* **267,** 1075–1086, doi: 10.1002/jmor.10461 (2006).

IX. Chen, P. Y., Stokes, A. G. & McKittrick, J. Comparison of the structure and mechanical properties of bovine femur bone and antler of the North American elk (*Cervus elaphus canadensis*). *Acta Biomater.* **5,** 693–706, doi: 10.1016/j.actbio.2008.09.011 (2009).

X. Landete-Castillejos, T. *et al.* Influence of physiological effort of growth and chemical composition on antler bone mechanical properties. *Bone* **41,** 794–803, doi: 10.1016/j.bone.2007.07.013 (2007).

XI. Currey, J. D. *Bones: Structure And Mechanics*. (Princeton University Press, 2002).

XII. MacGregor, A. G. & Currey, J. D. Mechanical properties as conditioning factors in *the* bone and antler industry of the 3rd to the 13th century AD, *J. Archaeol. Sci.* **10,** 71–77 (1983).

XIII. Chen, P. Y. *et al.* Structure and mechanical properties of selected biological materials. *J. Mech. Behav. Biomed. Mater.* **1,** 208–226, doi: 10.1016/j.jmbbm.2008.02.003 (2008).

XIV. Currey, J. D. *et. al.* The mechanical properties of red deer antler bone when used in fighting, *J. Exp. Biol.* **212,** 3985–3993, doi: 10.1242/jeb.032292 (2009).

XV. Currey, J. D. The effects of drying and re-wetting on some mechanical properties of cortical bone. *J. Biomech.* **21,** 439–441 (1988).

XVI. Reilly, D. T. & Burstein, A. H. The elastic and ultimate properties of compact bone tissue, *J. Biomech.* **8,** 393–405 (1975).

Supplementary Figure S4: X-ray diffractograms for *M. primigenius.*

a) M517

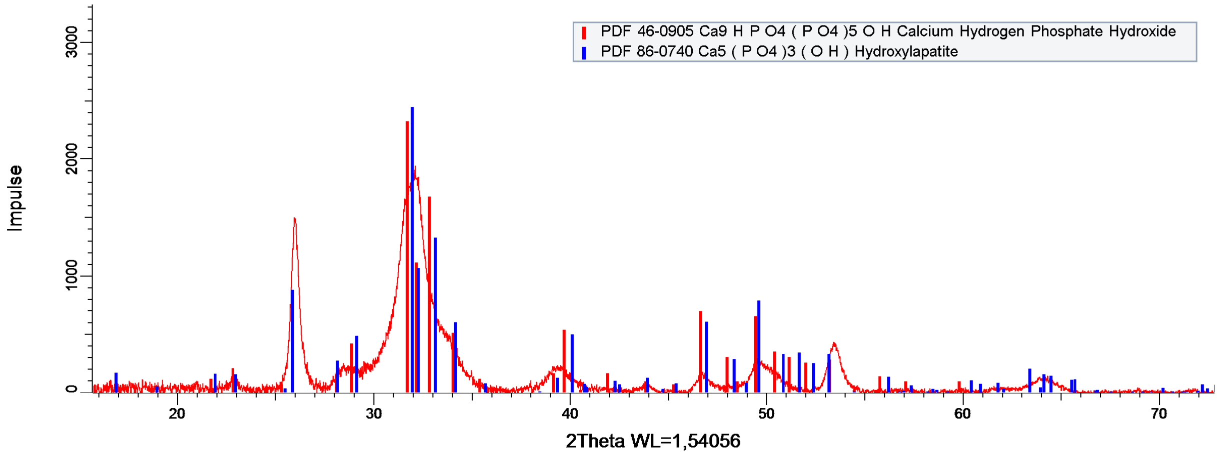

b) M512

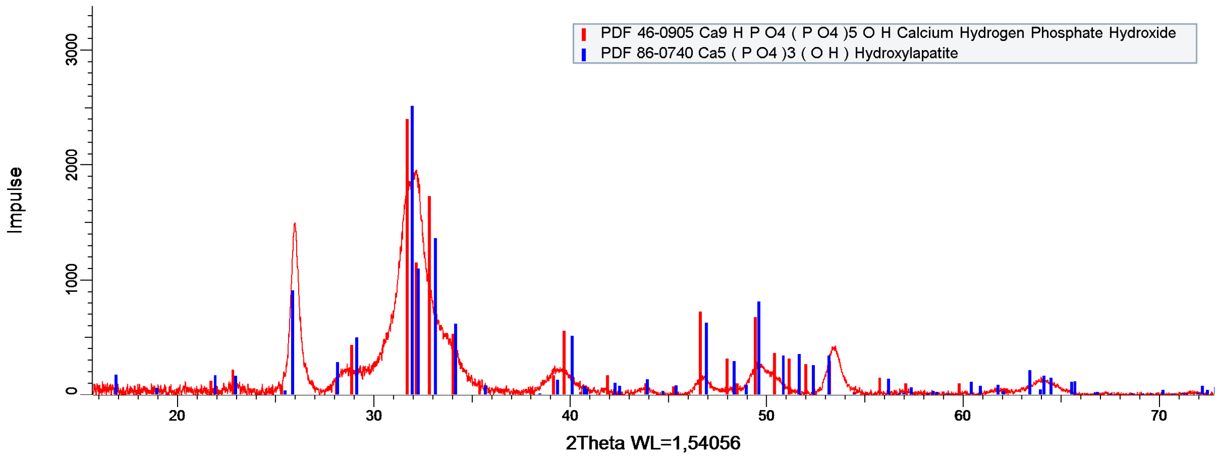

c) M516

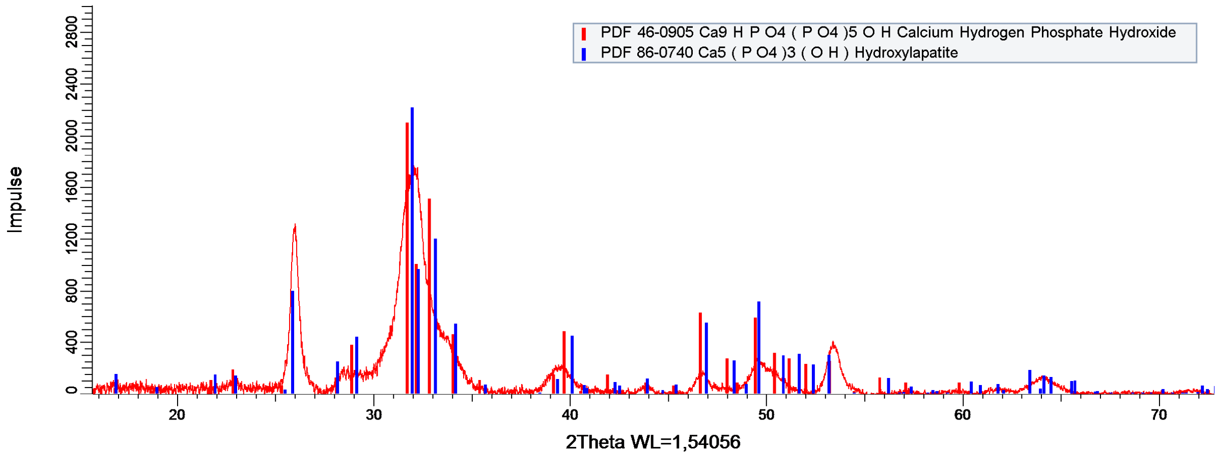

d) M515

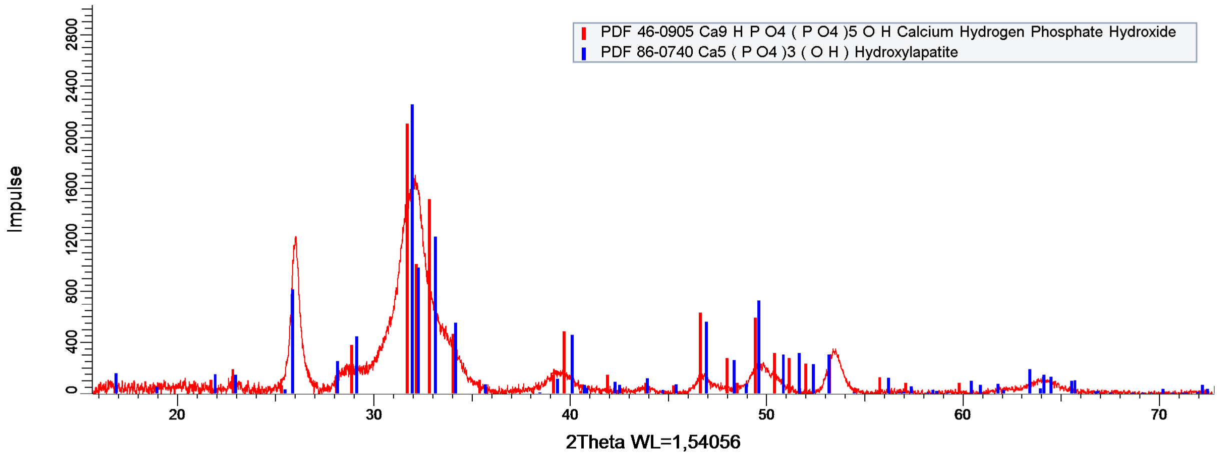
PDF 46-0905 Ca9 H P O4 (P O4)5 O H Calcium Hydrogen Phosphate Hydroxide
PDF 86-0740 Ca5 (P O4)3 (O H) Hydroxylapatite
Impulse
0
400
800
1200
1600
2000
2400
2800
20
30
40
50
60
70
2Theta WL=1,54056

e) M513

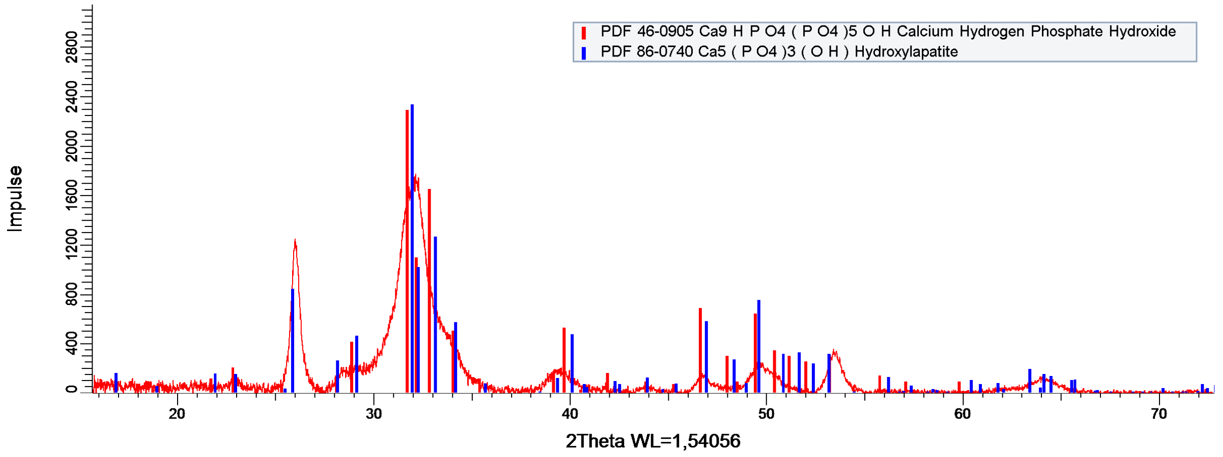
PDF 46-0905 Ca9 H P O4 (P O4)5 O H Calcium Hydrogen Phosphate Hydroxide
PDF 86-0740 Ca5 (P O4)3 (O H) Hydroxylapatite
Impulse
0
400
800
1200
1600
2000
2400
2800
20
30
40
50
60
70
2Theta WL=1,54056

Supplementary Figure S5: X-ray diffractograms for *L.africana*.

a) E3108

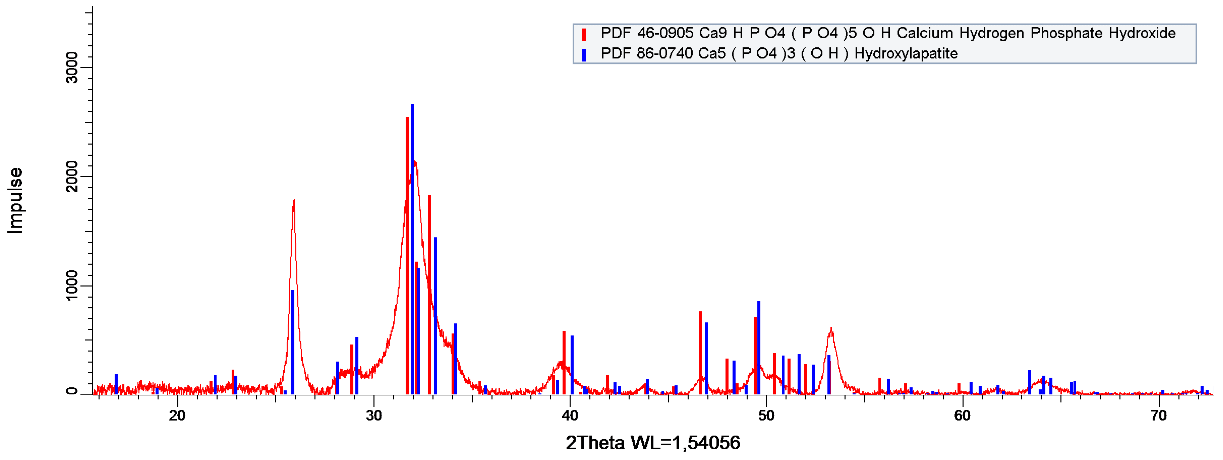

b) E3106

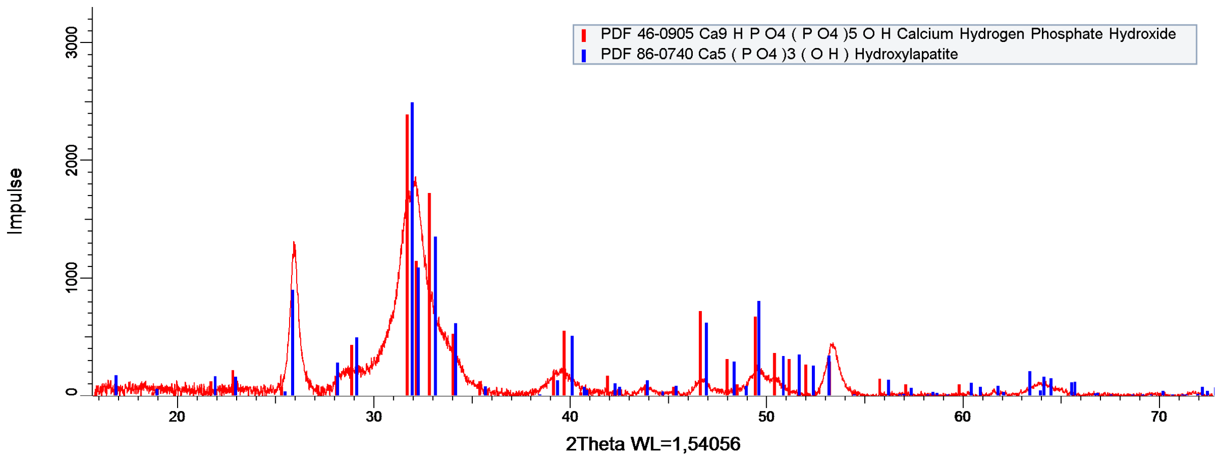

c) E3107

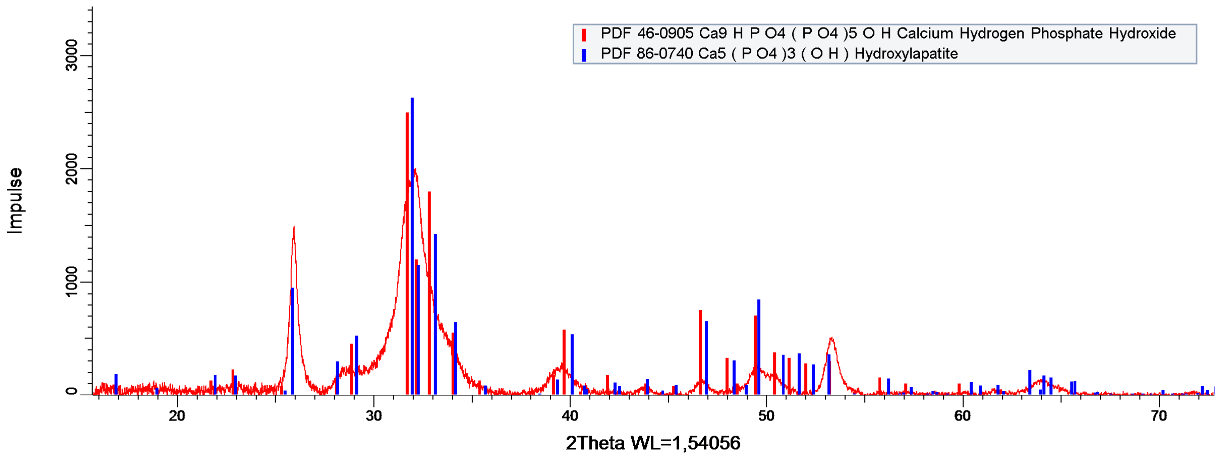

d) E3102

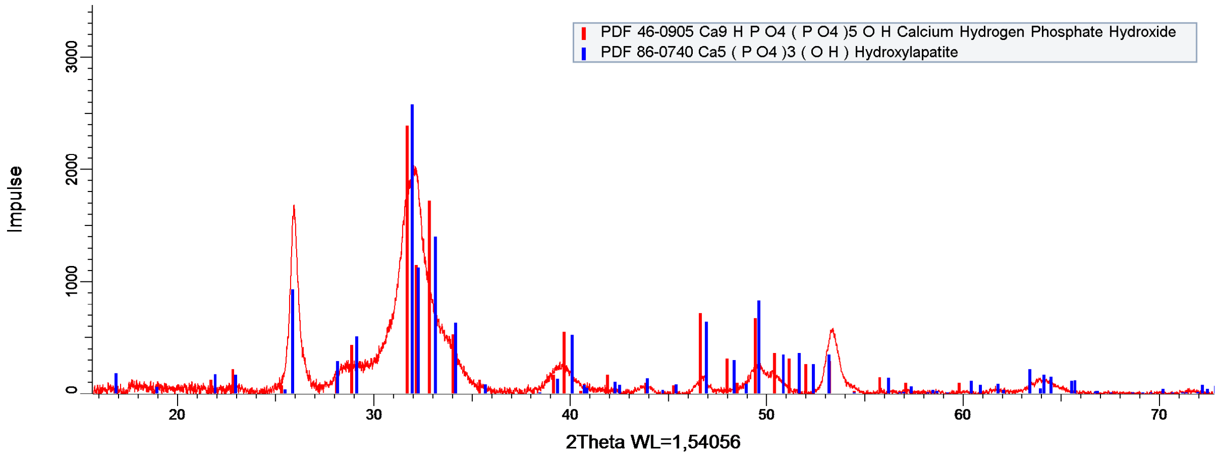

e) E3103

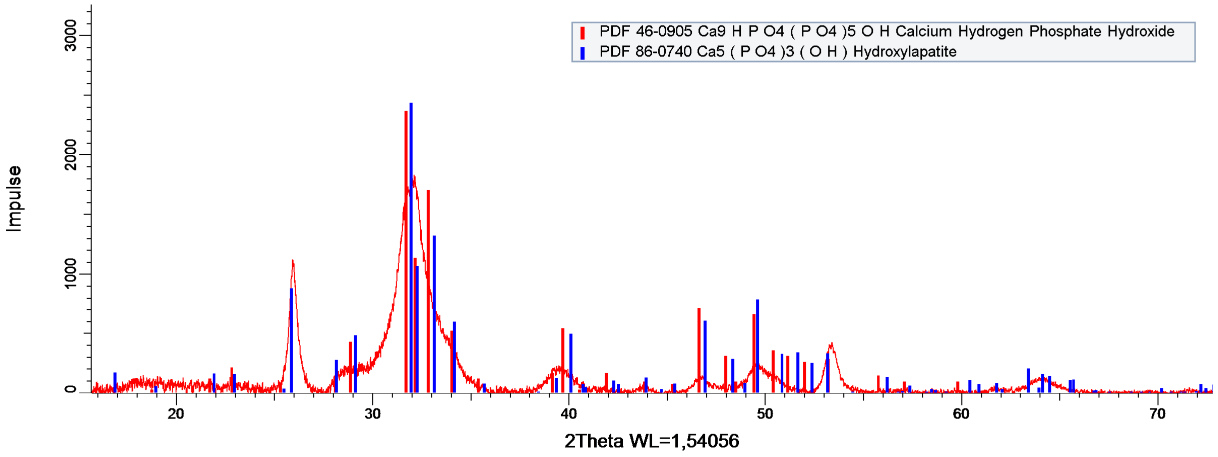

Supplementary Table S6: XRD results. Sample positions and positions of measurement marked in the Article Figs. 2a & c.

	**M512**	**M513**	**M515**	**M516**	**M517**	**E3102**	**E3103**	**E3106**	**E3107**	**E3108**
2θ [°]	26.010	26.000	26.040	26.005	25.960	25.940	25.920	25.935	25.940	25.945
FWHM [°]	0.465	0.480	0.475	0.470	0.460	0.460	0.440	0.460	0.435	0.410
FWHM [rad]	0.0081	0.0084	0.0083	0.0082	0.0080	0.0080	0.0077	0.0080	0.0076	0.0072
L (002) [nm]	21.712	20.779	21.265	21.383	21.778	21.684	22.932	21.661	23.396	25.478

	**$\bar{x}$ mammoth**	**σ mammoth**	**σ^2 mammoth**	**$\bar{x}$ elephant**	**σ elephant**	**σ^2 elephant**
2θ [°]	26.003	0.026	0.001	25.936	0.009	0.000
FWHM [°]	0.470	0.007	0.000	0.441	0.019	0.000
FWHM [rad]	0.0082	0.0001	0.0000	0.0077	0.0003	0.0000
L (002) [nm]	21.384	0.359	0.161	23.030	1.402	2.456
